# Supplementary material for: 3D-printed external cranial protection following decompressive craniectomy after brain injury: A pilot feasibility cohort study
Source: PLoS One. 2021 Oct 28;16(10):e0258296. doi: 10.1371/journal.pone.0258296 (PMC8553164; doi:10.1371/journal.pone.0258296)
Supplement: S1 File — (PDF) [file pone.0258296.s001.pdf]

AN EXPLORATORY STUDY ON THE DEVELOPMENT OF A NOVEL HEAD PROTECTION PROTOTYPE DEVICE FOR POST-DECOMPRESSION CRANIECTOMY PATIENTS

(NHG DSRB 2019/_00155)

DATA COLLECTION FORM LIST OF VARIABLES AFTER SCREENING

CASE NO: HP_00_______   INITIALS: ___________

DATA COLLECTION FORM

Filled by: Name _______________ Date: _____________Sign_________________

(A) Demographics and Clinical Characteristics

Age (in years): ______________

Gender: (1) Male (2) Female

Race: (1) Chinese (2) Malay (3) Indian (4) Others

Employed: (1) Yes (2) No

If Employed, Occupation: (1) Legislators/Senior Officials/Managers (2) Professional (3) Associate Professionals/Technician (4)Clerical support worker (5)Service/sales worker (6) Agricultural and Fishery Worker (7) Craftsmen/related trades worker (8) Plant/machine operators and assemblers (9) Cleaners/labourers (10) Others

Source of referral: (1) Restructured Hospital (2) Private hospital (3) Home/outpatient clinic

(4) International

Year of event: ______ Acute LOS (days): _____________ Rehabilitation LOS: _______________

Injury Type:

(1) Ischaemic Stroke (2) ICH (3)TBI (4) AVM (5) Aneurysmal SAH (6) Others _____________

Please proceed to Sections B and C if injury is a stroke (1.2)

Please proceed to section D if injury is Traumatic Brain Injury (3)

(B) Characteristic of stroke (1.2)

Stroke Type: (1) Cerebral Infarction (2) Intracerebral haemorrhage Specify_________

Side of stroke: (1) Right (2) Left (3) Bilateral

If Ischemic stroke, TOAST classification: (1) TACI (2) PACI (3) Brainstem (4) Lacunar

(C) Stroke severity

For Ischemic stroke, NIHSS on admission (at acute hospital): ___________________________

For Haemorrhagic stroke, GCS at A&E (3 to 15): ______________/15 WFNS (if SAH): _______

(D) Characteristics of Traumatic Brain Injury

Cause of traumatic brain injury: (1) Fall on level ground (2) Fall from height

(3) Motor vehicle accident (4)Personal mobility device related

(5) Assault (6) Sports (7) Others

Type of lesion: (1) Diffuse (2) Focal

If diffuse, type of injury: (1) diffuse axonal injury (2) concussion

If focal, type of injury: (1) penetrating injury (2) cerebral contusion

(3) intracerebral haemorrhage (4) epidural hematoma (5) subdural hematoma (6)SAH (7) IVH

(E) Severity of Traumatic Brain Injury

GCS on admission: _______________/15

Duration of loss of consciousness (days): _______________________

Emerged from PTA: (1) Yes :Duration PTA(days) ________ (2) No : Duration of PTA (days):______

(F) Decompressive Craniectomy (DC) Information

Number of DC surgeries for current event: ___________

Complications related to DC: (1) Yes (2) No

If Yes, Types of Complications:

(1) Wound breakdown (2) Wound Abscess (3) New collection/SDH

(4) Brain Abscess (5) Brain Herniation (6) Hydrocephalous

(7) Trephined Brain Syndrome (8) Others

Decision for Cranioplasty at time of recruitment: (1) Yes (2) No (3) Refused (4) Unknown

HPPD retained by Subject: (1) Yes (2) No

Date of cranioplasty: _____________

Type of Cranioplasty: (1) PEEK (2) Titanium (3)Acrylic (4) Others ___________________

(G) WEIGHT of HPPD : _______________g HPPD +straps: _____________g

(1) Week 0 Assessment (Date ___________________)

Filled by: Name _______________ Date: ____________Sign_________________

<u>Task List:</u>

DC pre-fit wound check Satisfactory: (1) Yes (2) No

HPPD Fits: (1) Yes (2)No Comments: ______________________

Strap Fit : (1) Yes (2) No Comments: ______________________

<u>Post-Fit 30 minutes Check:</u>

Subject pain rating: VAS 0 to 10: ________

Subject Comfort level: Least 0---------10 Most : _______

Early redness/skin reaction: (1) Yes (2) No

<u>Post Fit Education:</u>

Don and Doff training (1) Yes (2) No

Competency check Don/Doff (1) Yes (2) No

Explanation of Wearing Log (1)Yes (2) No

HPPD delivered to subject (1) Yes (2)No

Follow up Schedule given (1) Yes (2)No

"I acknowledge receipt of my customised Head Protection Prototype Device for wearing at home and that I have been educated on how to use it and chart its use"

___________/______________ ____________________________________

Name of Subject/LAR/Signature Name of Study Team Member/Signature

Date__________________________ Date______________________________

Follow Up Visit 1.1 (1st Phone visit) Day 1

Date: __________________(dd/mm/yyyy)   Interviewer:__________/Signature____
Time: ___________ (HH:MM)   Interviewee:_____________________

Have there been any new medical events or symptoms (e.g. falls, seizures) since the last session:
(1) Yes   (2) No
If yes, specify: ____

Time head protection was worn (in hours):
Indicate activity done next to the hours shaded

AM:

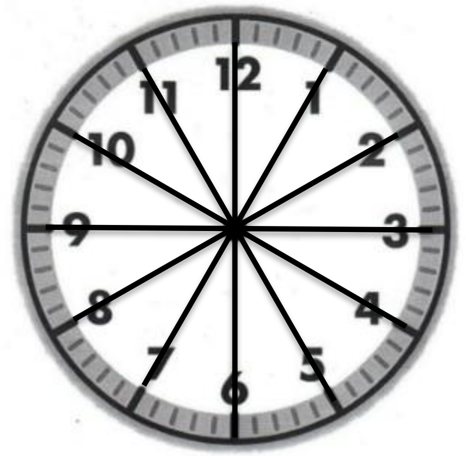

PM:

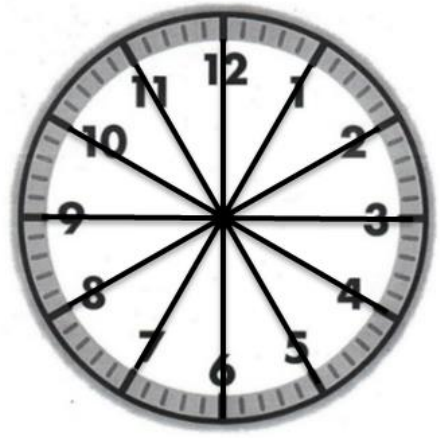

Pain: (1) Yes   (2) No
If yes, VAS score (0-10): _____________________________

Pressure: (1) Yes   (2) No
If yes, rate severity from 0-10 (0 being none and 10 being most severe): _______________

Itch: (1) Yes   (2) No
If yes, rate severity from 0-10 (0 being none and 10 being most severe): _______________

Wound changes: (1) Yes (2) No<br>If yes, specify:<br>(1) skin breakdown (2) skin infection (3) skin discharge (4) rashes on surrounding area outside surgical scar
Dislodgement: (1) Yes (2) No
Rate cosmesis from 0- 10 (0 being very poor appearance, 10 being very pleasing appearance):<br>_______

**Follow up Visit 1.2 (2nd Phone visit) : Day 3**
Date: __________________(dd/mm/yyyy)   Interviewer:__________/Signature____<br>Time: ___________ (HH:MM)   Interviewee:_____________________
Have there been any new medical events or symptoms (e.g. falls, seizures) since the last session:<br>(1) Yes   (2) No<br>If yes, specify: ____
Time head protection was worn (in hours):<br>Indicate activity done next to the hours shaded<br><br>AM:<br>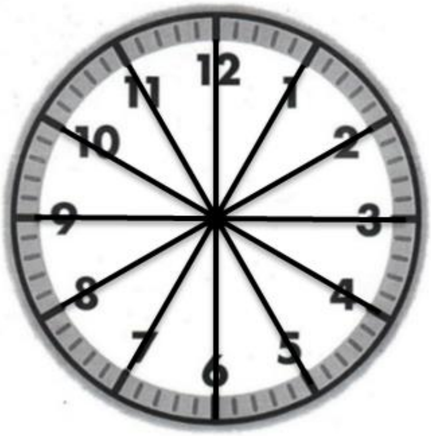<br>PM:<br>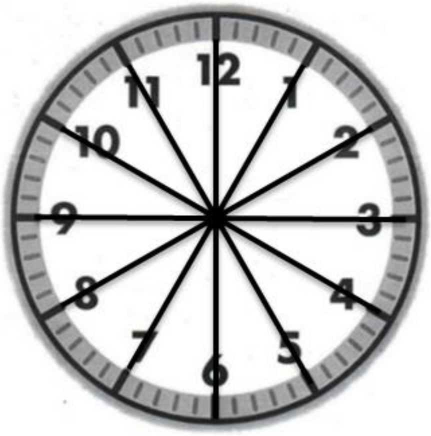
Pain: (1) Yes   (2) No<br>If yes, VAS score (0-10): ____________________________
Pressure: (1) Yes   (2) No<br>If yes, rate severity from 0-10 (0 being none and 10 being most severe): ______________
Itch: (1) Yes   (2) No<br>If yes, rate severity from 0-10 (0 being none and 10 being most severe): ______________

Wound changes: (1) Yes (2) No<br>If yes, specify:<br>(1) skin breakdown (2) skin infection (3) skin discharge (4) rashes on surrounding area outside surgical scar
Dislodgement: (1) Yes (2) No
Rate cosmesis from 0- 10 (0 being very poor appearance, 10 being very pleasing appearance): ______

(2) End of Week 1 Assessment

**Follow up Visit 2 (1st onsite visit)**

Date: __________________(dd/mm/yyyy) Interviewer:__________/Signature____
Time: ___________ (HH:MM) Interviewee:_____________________

Have there been any new medical events or symptoms (e.g. falls, seizures) since the last session:
(1) Yes (2) No
If yes, specify: ____

Time head protection was worn (in hours):
Indicate activity done next to the hours shaded

AM:

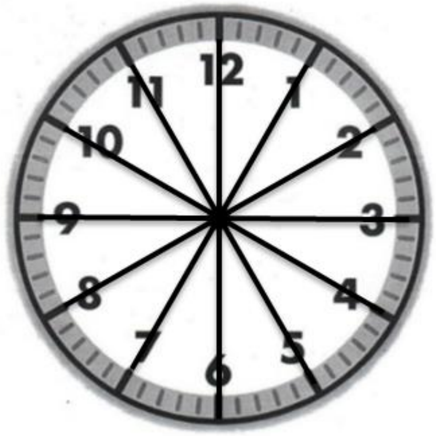

PM:

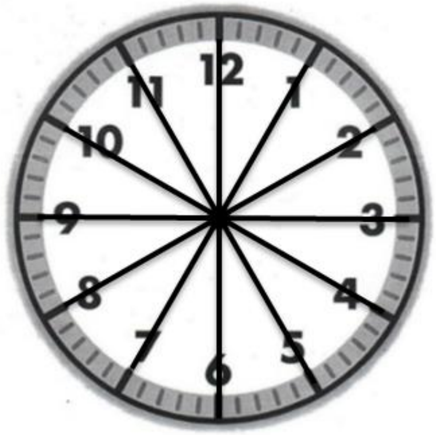

Pain: (1) Yes (2) No
If yes, VAS score (0-10): _____________________________

Pressure: (1) Yes (2) No
If yes, rate severity from 0-10 (0 being none and 10 being most severe): _______________

Itch: (1) Yes (2) No
If yes, rate severity from 0-10 (0 being none and 10 being most severe): _______________

Wound changes: (1) Yes (2) No<br>If yes, specify:<br>(1) skin breakdown (2) skin infection (3) skin discharge (4) rashes on surrounding area outside surgical scar
Dislodgement: (1) Yes (2) No
Rate cosmesis from 0- 10 (0 being very poor appearance, 10 being very pleasing appearance):<br>______

(3) End of Week 2 assessment

**Follow up Visit 3 (2nd onsite visit)**	
Date: __________________(dd/mm/yyyy)<br>Time: ___________ (HH:MM)	Interviewer:__________/Signature____<br>Interviewee:____________________
Have there been any new medical events or symptoms (e.g. falls, seizures) since the last session:<br>(1) Yes (2) No<br>If yes, specify: ____	
Time head protection was worn (in hours):<br>Indicate activity done next to the hours shaded<br><br>AM:<br>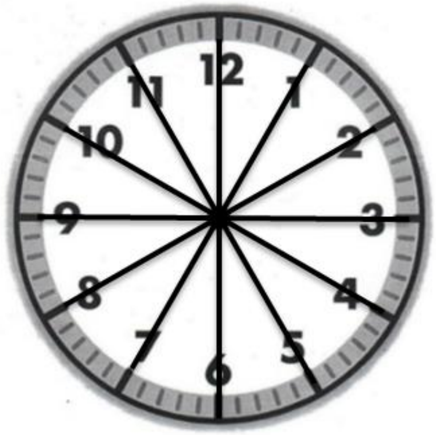<br>PM:<br>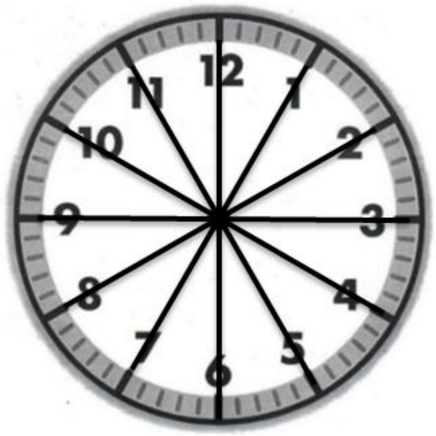	
Pain: (1) Yes (2) No<br>If yes, VAS score (0-10): ____________________________	
Pressure: (1) Yes (2) No<br>If yes, rate severity from 0-10 (0 being none and 10 being most severe): ______________	
Itch: (1) Yes (2) No<br>If yes, rate severity from 0-10 (0 being none and 10 being most severe): ______________	

Wound changes: (1) Yes (2) No<br>If yes, specify:<br>(1) skin breakdown (2) skin infection (3) skin discharge (4) rashes on surrounding area outside surgical scar
Dislodgement: (1) Yes (2) No
Rate cosmesis from 0- 10 (0 being very poor appearance, 10 being very pleasing appearance): _______

(4) End of Week 4 assessment

**Follow Up Visit 4 (3rd onsite visit)**
Date: __________________(dd/mm/yyyy) Interviewer:__________/Signature____<br>Time: ___________ (HH:MM) Interviewee:____________________
Have there been any new medical events or symptoms (e.g. falls, seizures) since the last session:<br>(1) Yes (2) No<br>If yes, specify: ____
Time head protection was worn (in hours):<br>Indicate activity done next to the hours shaded<br><br>AM:<br>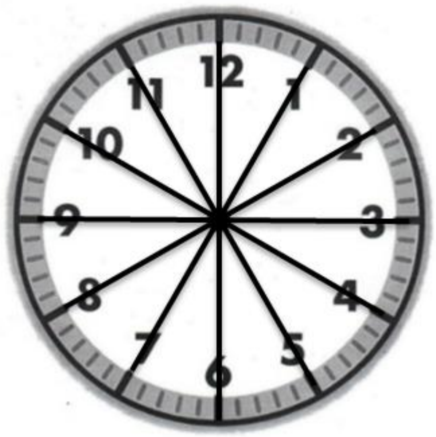<br>PM:<br>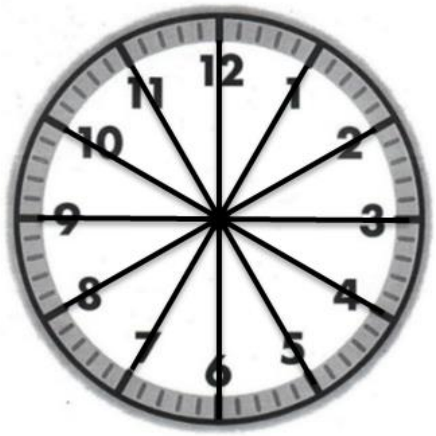
Pain: (1) Yes (2) No<br>If yes, VAS score (0-10): ____________________________
Pressure: (1) Yes (2) No<br>If yes, rate severity from 0-10 (0 being none and 10 being most severe): ______________
Itch: (1) Yes (2) No<br>If yes, rate severity from 0-10 (0 being none and 10 being most severe): ______________

Wound changes: (1) Yes (2) No<br>If yes, specify:<br>(1) skin breakdown (2) skin infection (3) skin discharge (4) rashes on surrounding area outside surgical scar
Dislodgement: (1) Yes (2) No
Rate cosmesis from 0- 10 (0 being very poor appearance, 10 being very pleasing appearance): ______

(5) End of Week 6 assessment

**Follow Up Visit 4.5 (3rd Phone visit)**
Date: __________________(dd/mm/yyyy) Interviewer:__________/Signature____<br>Time: ___________ (HH:MM) Interviewee:____________________
Have there been any new medical events or symptoms (e.g. falls, seizures) since the last session:<br>(1) Yes (2) No<br>If yes, specify: ____
Time head protection was worn (in hours):<br>Indicate activity done next to the hours shaded<br><br>AM:<br>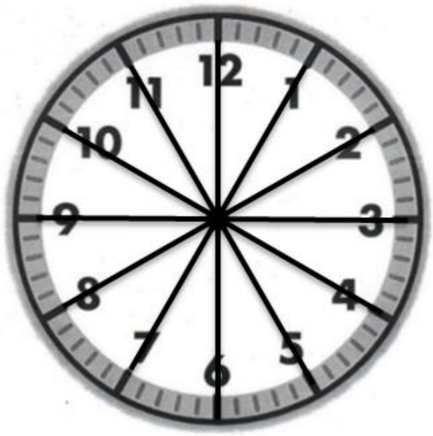<br>PM:<br>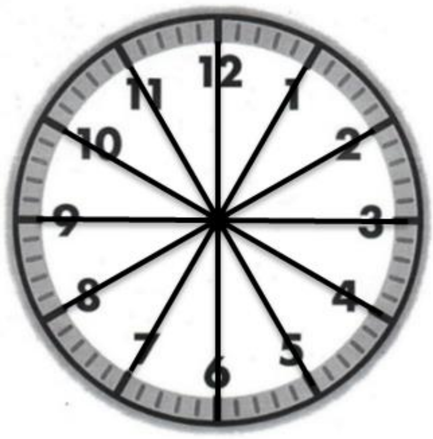
Pain: (1) Yes (2) No<br>If yes, VAS score (0-10): ____________________________
Pressure: (1) Yes (2) No<br>If yes, rate severity from 0-10 (0 being none and 10 being most severe): ______________
Itch: (1) Yes (2) No<br>If yes, rate severity from 0-10 (0 being none and 10 being most severe): ______________

Wound changes: (1) Yes (2) No<br>If yes, specify:<br>(1) skin breakdown (2) skin infection (3) skin discharge (4) rashes on surrounding area outside surgical scar
Dislodgement: (1) Yes (2) No
Rate cosmesis from 0- 10 (0 being very poor appearance, 10 being very pleasing appearance): _______

(6) End of Week 8 assessment (Final assessment)

**Visit 5 (4th onsite visit)**
Date: ___________________(dd/mm/yyyy) Interviewer:__________/Signature____<br>Time: ____________ (HH:MM) Interviewee:_____________________
Have there been any new medical events or symptoms (e.g. falls, seizures) since the last session:<br>(1) Yes (2) No<br>If yes, specify: ____
Time head protection was worn (in hours):<br>Indicate activity done next to the hours shaded<br><br>AM:<br>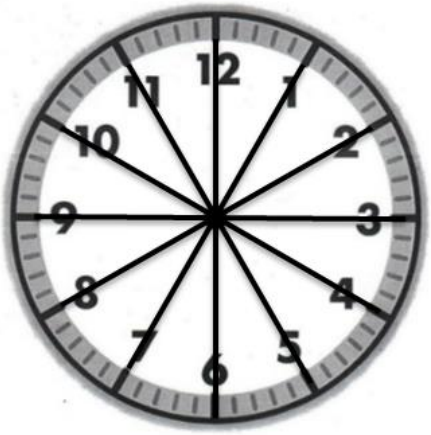<br>PM:<br>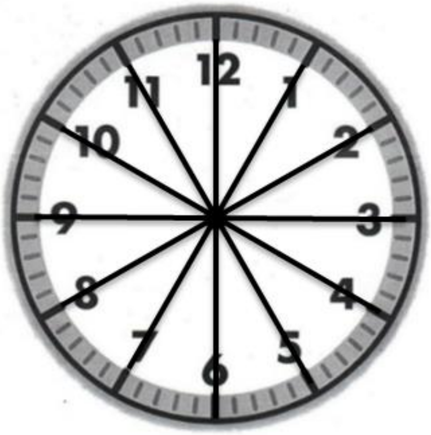
Pain: (1) Yes (2) No<br>If yes, VAS score (0-10): ______________________________
Pressure: (1) Yes (2) No<br>If yes, rate severity from 0-10 (0 being none and 10 being most severe): _______________
Itch: (1) Yes (2) No<br>If yes, rate severity from 0-10 (0 being none and 10 being most severe): _______________

Wound changes: (1) Yes (2) No<br>If yes, specify:<br>(1) skin breakdown (2) skin infection (3) skin discharge (4) rashes on surrounding area outside surgical scar
Dislodgement: (1) Yes (2) No
Rate cosmesis from 0- 10 (0 being very poor appearance, 10 being very pleasing appearance): ______

-END OF ASSESSMENT-
