# Supplementary material for: 3D-printed external cranial protection following decompressive craniectomy after brain injury: A pilot feasibility cohort study
Source: PLoS One. 2021 Oct 28;16(10):e0258296. doi: 10.1371/journal.pone.0258296 (PMC8553164; doi:10.1371/journal.pone.0258296)
Supplement: S3 File — (PDF) [file pone.0258296.s003.pdf]

STUDY PROTOCOL

PROTOCOL TITLE:

AN EXPLORATORY STUDY ON THE DEVELOPMENT OF A NOVEL HEAD PROTECTION PROTOTYPE DEVICE FOR POST-DECOMPRESSION CRANIECTOMY PATIENTS

PROTOCOL NUMBER:

DSRB 00155/2019

PROTOCOL VERSION: Version 5
PROTOCOL DATE: 22 Aug 2020

PRINCIPAL INVESTIGATOR:

Dr CHUA Sui Geok, Karen. Senior Consultant, TTSH Rehabilitation Medicine

SITE PRINCIPAL INVESTIGATOR:

Dr RAO, Jai, National Neuroscience Institute, NNI Neurosurgery

Co- INVESTIGATORS :

Dr Rathi D/O Ratha Krishnan, TTSH Rehabilitation Centre (TTSH Rehabilitation Centre)
Dr Yen Jia Min (c/o TTSH Rehabilitation Centre)
Ms Tegan K Plunkett (TTSH Rehabilitation Centre)
Ms Soh Yan Ming (TTSH Rehabilitation Centre)
Ms Ng Suan Gek (NNI Neurosurgery)

STUDY SITES:

TTSH Rehabilitation Centre @AMKH, 17 Ang Mo Kio Ave 9, Singapore 569766.
TTSH CART clinic 5B, 11 Jln Tan Tock Seng, Singapore 308433

COLLABORATORS: (Technical vendor)

Mr Looi Jun Cong, Sean
Ms Catherine Chia

Company Name: Creatz3D,
Address: 180 Paya Lebar Road, #08-07 Yi Guang Factory Building. Singapore 409032.

TABLE OF CONTENTS

1. BACKGROUND AND RATIONALE 4

1.1. General Introduction 4
1.2. Rationale and justification for the Study 5
a. Rationale for the Study Purpose 5
b. Rationale for Doses Selected 6
c. Rationale for Study Population 6
d. Rationale for Study Design 6

2. HYPOTHESIS AND OBJECTIVES 6

2.1. Hypothesis 7
2.4. Potential Risks and benefits: 7
a. End Points - Efficacy 7
b. End Points - Safety 7

3. STUDY POPULATION 5

3.1. List the number of subjects to be enrolled. 7
3.2. Criteria for Recruitment 8
3.3. Inclusion Criteria 8
3.4. Exclusion Criteria 8
3.5. Withdrawal Criteria 9
3.6. Subject Replacement 9

4. TRIAL SCHEDULE 9

5. STUDY DESIGN 6

5. STUDY DESIGN 10
5.1. Summary of Study Design 10

6. METHODS AND ASSESSMENTS 10

6.1. Randomisation and Blinding 10
6.2. Contraception and Pregnancy Testing 10
6.3. Study Visits and Procedures 10

7. TRIAL MATERIALS 12

7.1. Trial Product (s) 12
7.2. Storage and Drug Accountability 12

8. TREATMENT 13

8.1. Rationale for Selection of Dose 13
8.2. Study Drug Formulations 13
8.3. Study Drug Administration 13
8.4. Specific Restrictions / Requirements 13
8.5. Blinding 13
8.6. Concomitant therapy 13

9. SAFETY MEASUREMENTS 13

9.1. Definitions 13
9.2. Collecting, Recording and Reporting of "Unanticipated Problems Involving Risk to Subjects or Others" – UPIRTSO events to the NHG Domain Specific Review Boards (DSRB) 13

9.3. COLLECTING, RECORDING AND REPORTING OF SERIOUS ADVERSE EVENTS (SAEs) TO THE HEALTH SCIENCE AUTHORITY (HSA) 14
9.4. SAFETY MONITORING PLAN 15
9.5. COMPLAINT HANDLING – 15

10. DATA ANALYSIS 15

10.1. DATA QUALITY ASSURANCE 15
10.2. DATA ENTRY AND STORAGE 15

11. SAMPLE SIZE AND STATISTICAL METHODS 15

11.1. DETERMINATION OF SAMPLE SIZE 15
11.2. STATISTICAL AND ANALYTICAL PLANS 15

12. ETHICAL CONSIDERATIONS 16

12.1. INFORMED CONSENT 16
12.2. IRB REVIEW 16
12.3. CONFIDENTIALITY OF DATA AND PATIENT RECORDS 16

13. PUBLICATIONS 16

14. RETENTION OF TRIAL DOCUMENTS 17

1. BACKGROUND AND RATIONALE

Annually, approximately 80-100 patients within NNI and TTSH public hospitals with severe stroke, traumatic brain injury (TBI) and spontaneous subarachnoid haemorrhage (SAH) undergo unilateral or bilateral wide decompressive craniectomy (DC) due to raised intracranial pressures. Such life-saving surgeries leave survivors with a sunken skull bone flap in the recovery phase. Following subacute inpatient rehabilitation, ~50%-75% of patients regain locomotor abilities and are ambulant with varying degrees of assistive aid. Upon discharge, most are able to further improve to supervision or modified independence functional mobility levels in the community.

As a result of residual stroke or TBI related residual motor, balance and / or sensory weakness, cognitive and behavioral impairments, impulsivity, these patients are identified to have significantly increased fall risk. Furthermore, the unprotected cerebrum post DC is particularly vulnerable to secondary injury from minor falls, in addition to long-term post DC complications such as flap herniation through the bony skull defect, hydrocephalous and syndrome of the trephined brain. Definitive elective cranioplasty either with artificial materials or autologous bone flap provides the only solution to these poorly understood conditions.

An average 3 -6 months' interval from acute craniectomy to definitive cranioplasty is common due to several factors. These include time needed for spontaneous neurological recovery, resolution of acute neurosurgical and medical complications, healing of the craniectomy wound and completion of inpatient rehabilitation (mean rehabilitation length of stay @TTSH Rehabilitation Centre ~30 days). Other factors include individual neurosurgeons' practice, patient/family consent, implant selection and fabrication time.

During this period, there is no protection for the skull defect. While general guidelines agree that helmet wear is mandatory from the early phase of recovery and during ambulation to protect the skull flap, there is no agreed protocol for post DC cranial defect management and often poor compliance to protective helmets exists. Standard measures include off the shelf bicycle or riding helmets ($30-$40 each) or ventilated and rigid head protection helmets (~$300-500 each), are uncomfortable in Singapore's tropical climate and need to be individually imported. The latter are currently used at TTSH Rehabilitation Centre for mobile patients undergoing ambulation training or who are independent. However, they are hot in our tropical weather, heavy, uncomfortable and are socially not acceptable with poor cosmesis to recovering patients who may have physical and functional impairments, hence compliance is extremely low and the large majority of patients awaiting elective cranioplasty are observed wearing caps, hats or head scarves rather than head protection helmets in the post-discharge phase while awaiting cranioplasty.

While the AHA/ASA concludes that DC is a life-saving procedure, little is written in the literature about the optimal care management of the skull defect in the post DC phase. (Livesay S 2011)

1.1. General Introduction

Decompressive Craniectomy (DC) is a surgical procedure that involves removing a portion of the skull and closing the scalp without reimplantation of the bone, commonly as a life-saving surgery after

malignant cerebral infarctions, intracerebral hemorrhages and severe traumatic brain injury. This results in a cranial defect. Craniectomy helps to increase the buffering capacity of the cranium, allows outward herniation of brain tissue and reduces intracranial pressure. (Mayfieldclinic.com/PE-TBI.htm) (www.jpma.org.pk/full_article_text.php?article_id=3932)

Though the procedure is technically straightforward, serious complications, apart from wound infections, have been reported in the acute and chronic phase. These include herniation of the cerebral cortex through bone defect, subdural effusion, seizures, hydrocephalus and syndrome of the trephined. About half of the patients in a study by Honeybul et al in 2011, had at least one complication following craniectomy. It was found that the presence of at least one complication significantly doubled the length of stay in the hospital or rehabilitation facility. (https://doi.org/10.1089/neu.2010.1612).

In a literature review of more than 60 publications, patients post DC, were identified to be high fall risk and at high risk of injury. Fall prevention and protection of exposed cranium from fall or pressure were considered important in the first few weeks post craniectomy. (Livesay S 2014).
Post DC patients are deemed to be lacking optimal coordination and balance and run the risk of trauma to the unprotected brain should they fall (Bostrom S, 2005).
In a separate study, it was recommended that post craniectomy patients should be considered high risk and institutions should establish or review their current management guidelines. (Honeybul S 2009). It was also recommended that falls risk assessment be done in this group of patients and helmet use be made mandatory in rehabilitative exercises (Bostrom S 2005)

Despite this, secondary traumatic brain injury following DC has rarely been reported. A solitary case report from a Perth hospital) documented a post-DC well-recovering patient after initial head trauma who suffered a fall on the bony defect and subsequently died from this secondary cerebral trauma. This underpins the importance of adequate preventive head protection for all DC patients. (Honeybul S 2009)

1.2. Rationale and justification for the Study

Currently, there exists no locally produced, viable, customized-skull defect protective external prostheses that is available to post DC patients in the recovery phase. A recently launched overseas prototype is referenced. (www.anatomics.com)

A proof-of-concept (POC) proposal to study the feasibility of customized head protection prototype device (HPPD) using 3D printed externally-applied prostheses integrated to the craniectomy bony skull defect. The proposed material is a bio-compatible, light-weight rigid /semi rigid material which can be fitted to the skull defect and interfaced with the subject's bony rim using soft, deformable material (e.g. silicon) and attached using an elastic head band. The prosthesis is removable for skin/prosthesis cleansing.

Our hypothesis is that the fabricated HPPD will be fitted feasibly and undergo a proof of concept trial for safety and wearability with systematic monitoring.

a. Rationale for the Study Purpose

There has to date, been only 1 publication concerning a novel method described by S. Bostrom (2005)

et al in a Swedish hospital where the doctors used methyl metacrylate (a type of bone cement in joint replacement surgery) to form an acrylic flap to cover the whole craniectomy site. The flap was created immediately post craniectomy, and was roughly up-sized to 12x13 centimetres to cover the bony defect and fixed to the skull with microplates temporarily until final cranioplasty was achieved with the patient's own bone flap. However, this procedure necessitated the creation of a larger skin dissection to cover the enlarged acryl flap and prolonged the operation time by 40 minutes. In this study, 4 patients successfully underwent this temporary invasive procedure. Though this method attempted to address the short comings of helmet wearing, it was invasive, not fully customised and carried with it, the risks of prolonged surgical and anaesthetic times. (Bostrom 2005)

Though mandatory helmet use post DC has been recommended during rehabilitation exercises, the uptake is low in the local rehabilitation setting. (Bostrom S 2005) The common reasons given by patients are discomfort of helmet use in the local tropical climate, high cost of a rigid protective appropriate- sized helmet given the relatively short time prior to cranioplasty and poor aesthetic appeal and social acceptability of the helmet. For off-the-shelf helmets, the situation is similar and an optimal fit and contour may not be possible

Hence, this necessitates a lighter, aesthetically appealing protective head protection device for a post DC patient in order to prevent secondary brain injury in the event of a fall during the recovery phase.

Hence, our study hopes to craft a lightweight non-invasive external prosthesis that is customised to the defect's size. The customised head protection prototype device (HPPD) it thus proposed with the added benefit of being detachable for hygiene purposes.

b. Rationale for Doses Selected

Not applicable

c. Rationale for Study Population

Subjects with acute or chronic skull defects as a result of surgical decompressive craniectomy (DC) who have refused, are not medically fit or are waiting for definitive cranioplasty.

d. Rationale for Study Design

As this is a first time in human study, the study design is exploratory open label pilot study with independent assessor.

2. HYPOTHESIS AND OBJECTIVES

The objectives of this exploratory feasibility study are to testbed first time in human, customized 3D printed fabricated prostheses by additive manufacturing using biocompatible materials in suitable subjects with a post decompressive craniectomy skull defect.

2.1. Hypothesis

Our primary hypothesis is that 80% of subjects will be safely fitted with head protection prototype devices (HPPD) without immediate complaints of pain or discomfort or signs of bony defect scar pressure or redness or wound breakdown within the first 2 hours in the research clinic.
Our secondary hypothesis is that 70% of these subjects will be without complaints of pain or discomfort or signs of bony defect scar pressure or redness or wound breakdown in the first week of outpatient monitoring.
In addition, subjects will tolerate HPPD wear progressively up to 8 weeks for specified high risk activities.
We anticipate a 25% dropout rate at 8 weeks.

2.2. Primary Objectives

These are to (1) test the feasibility and safety of customised 3D printed HPPD,
2) integrate the prostheses to the skull defect in removable manner and discharge patient with appropriate education, (3) to systematically monitor subjects for symptoms, compliance, complications and subjective feedback during the outpatient phase where progressive wear of HPPD and monitoring of acceptability will be monitored.

2.3. Secondary Objectives

As the grant duration is a short 12 months, secondary objectives will not be hypotheses-driven.
These may include more widespread multi-centre use of the HPPD if proven successful in this pilot and serve as a prototype for further improvement and development as a new collaborative service incorporating the HPPD occurring early after craniectomy.

2.4. Potential Risks and benefits:

a. End Points - Efficacy

Successful fabrication and integration of HPPD and feasibility of clinical use by subjects in terms of usability and lack of complications.

b. End Points - Safety

These include:
(i) Unable to fit subject safely with regards to wounds.
(ii) Failure to fabricate a usable or safe HPPD.
(iii) Skin allergy or reaction or pressure related complications to the HPPD or attachment straps

3. STUDY POPULATION

3.1. List the number of subjects to be enrolled.

Number of subjects to enroll: 10 (5 to 9 from TTSH and 1 to 5 from NNI)

Study subjects will be drawn from inpatient ward admissions from NNI and TTSH Rehabilitation Centre wards and TTSH rehabilitation outpatient clinics @AMKH and TTSH CART 5B via face to face contact. Permission will be obtained from primary physicians.

3.2. Criteria for Recruitment

Clinical assessment for eligibility will follow strict adherence to the screening protocol. Study team will determine suitability for recruitment based on the following criteria. There are no subject recruitment restrictions based on age, gender or race.

3.3. Inclusion Criteria

All subjects must meet all of the inclusion criteria to participate in this study.

1. Age 21 to 80 years, both males and females.
2. Presence of surgical unilateral or bilateral craniectomy performed for reasons of ischaemic or haemorrhagic stroke, Subarachnoid haemorrhage (SAH), traumatic brain injury (TBI), benign cerebral tumours, etc.
3. Stroke, SAH or TBI are diagnosed by specialists and confirmed on brain imaging studies (CT, MRI)
4. Duration from event > 30 days and either during inpatient or outpatient phase.
5. Presence of at least 1 post decompressive craniectomy CT brain film performed at NNI/TTSH.
6. Healed craniectomy surgical wound without bulging skin flap or active skin infection.
7. Patients awaiting elective cranioplasty or those who refuse cranioplasty.
8. Ability to understand simple instructions.
9. Presence of family members or NOK who can supervise the patient to don the head protection device, care for the material and regularly monitor for compliance and complications.
10. Reproductive age females should not be pregnant at the point of consent-taking and during the study.

Exclusion Criteria

All subjects meeting any of the exclusion criteria at baseline will be excluded from participation.

1. Patient in vegetative or minimally responsive state.
2. Presence of uncontrolled medical condition (uncontrolled hypertension, Diabetes Mellitus, sepsis or delirium, active malignancy either cranial or extracranial sites)
3. Presence of end organ failure (end stage renal or liver failure, renal dialysis, life expectancy <6 months)
4. Presence of pregnancy or lactation.
5. Presence of severe agitation /behavioural/active depression or anxiety/ drug or alcohol addiction which would negatively affect compliance,
6. Presence of unhealed head wound, active wound infection, scalp dermatitis, wound breakdown which would be worsened by pressure from the HPPD.
7. Presence of known allergy to the investigational products which is the 3D printed material (e.g. Nylon).
8. Subjects' CT brain imaging films are not available to the study team.

9. Absence of NOK who can assist monitoring unless subject is able to self-monitor.

3.4. Withdrawal Criteria

Reasons for discontinuation of study intervention/product:

1. Completion of all study interventions and visits.
2. Failure to fit suitable HPPD.
3. Development of allergic reactions deemed due to the HPPD.
4. Development of new surgical scar infection deemed related or unrelated to the HPPD.
5. Unrelated medical or neurosurgical complications which alter eligibility to participate in the study.
6. Failure of study sponsor or vendor providing HPPD.
7. Study closure due to DSMB review.

3.5. Subject Replacement

Subjects who drop out will not be replaced.

4. TRIAL SCHEDULE

1. Obtainment of ethical approvals from Domain Specific Review Boards (DSRB)
2. Signed research collaborative agreements between TTSH and NNI and separate service agreement with technical vendor.
3. Setting up of research accounts and codes and clinic code.
4. Clinical trial commences with face to face identification of suitable subjects from TTSH and NNI (TTSH) acute /rehabilitation wards and TTSH outpatient clinics.
5. Contact, eligibility screening, selection of eligible patients and informed consent process at the research sites at TTSH rehabilitation centre wards or clinics and /or TTSH-CART clinic 5B and subject will be enrolled on CDOC and assigned research code number.
6. NNI Neurosurgeon of study team will be informed of enrolled subjects' details to select suitable CT brain images.
7. These images will be sent to NNI Neuroradiology service staff for anonymisation of the selected CT brain scans.
8. Anonymised CT brain scans will be stored on CD-ROMS and labelled with assigned research code.
9. These will be transmitted to the vendor for segmentation and 3D printing.
10. 3D printed skull replacement piece will be labelled with research code and delivered in a sealed box to TTSH CART research site/TTSH Rehabilitation Centre and handed to a member of the study team for safe keeping under lock and key.
11. Research subject till be contacted for on-site physical visit for customisation of prosthesis fit with attachment of silicone padding to bony rim.
12. Assessment of fit on subject's skull by team and comfort of patient for first 30 minutes of donning.
13. If fit is deemed by study team to be not suitable, further 3D printing iterations as needed to improve customisation.
14. If suitable fit, integration to bony flap of patient with Velcro straps, elastic bands or caps.
15. Subject is monitored for first 30 minutes, educated by study coordinator or nurse in research clinic

site on donning, doffing, wearing schedule log, complications to look out for, monitoring and study visit schedule.

16. Subject brings HPPD home with supervision of NOK.
17. Outpatient monitoring phase for up to 8 weeks. (Follow up physical visits x 4 and phone calls x 3). If consented, photograph or videography may be used.
18. End of study at week 8 or earlier if has undergone cranioplasty.
19. Subject has option to retain his own HPPD if is comfortable wearing, has no adverse events, and is still awaiting or has refused cranioplasty.

5. STUDY DESIGN

5.1. Summary of Study Design

Study design is a pilot open label study with assessor measured outcomes. This study design is appropriate for the exploratory proof of concept study.

6. METHODS AND ASSESSMENTS

1. Proof of Concept with open label pilot feasibility clinical trial.
2. Number of subjects to enrol: 10 (5 to 9 from TTSH and 1 to 5 from NNI)
3. Outpatient monitoring phase for up to 8 weeks (1 screening, 1 fitting, 4 follow up visits and 3 phone visits]
4. If planned cranioplasty is performed within 8 weeks of study inclusion, subject involvement will end on date of scheduled cranioplasty.
5. Assessments involve subjective evaluation of suitability of fit and complications during visits, patient and NOK log of duration of wearing and subjective complaints.
6. Digital photographs with pixilation of facial features or short video clips to document fit or outcome will be taken if subject consents. These may be used for education or safety review of the investigational product

6.1. Randomisation and Blinding

As this is a feasibility pilot study using open label study design, there will no randomisation or blinding.

6.2. Contraception and Pregnancy Testing

Reproductive age females should not be pregnant at the point of consent-taking and during the study.

6.3. Study Visits and Procedures

Enrolled subjects will be required to visit the research site a total of ~6 times for 1.5 to 2 hours each time and undergo all trial procedures as stated below. (1 screening, 1 fitting, 4 follow up visits and 3 phone visits.

a. Screening Visits and Procedures

Visit 0: Screening for eligibility, explanation of clinical trial and informed consent process by patients/patient's LAR.

b. Study Visits and Procedures

Upon signing of informed consent, subject is recruited and enrolled on CDOC, the following processes will happen:

1. Identification and selection of suitable CT brain images (NNI Neurosurgeon)
2. Transmission of selected CT brain images to vendor for segmentation, processing, and 3D printing and packaging.
3. Delivery by vendor to research site (NNI/TTSH) and if preliminary fit is optimal, integration to subject skull defect with silicone padding and strapping to subject's scalp.
4. If non-optimal fit to patient as deemed by discomfort or observation of pressure signs after 30 minutes, further 3D printing iterations will be needed.
5. Subject education on donning/doffing, assessment of fit, cleansing, progressive wearing schedule and monitoring logs and study visit schedule as below.

   Study visit schedule:
   (Total of 6 physical visits (1 screening visit, 1 fitting visit, 4 follow up visits) and 3 phone call follow ups)

Visit 0: Screening for eligibility and informed consent process from patients'/patients' LAR.

Visit 1 (week 0): Fitting of HPPD and integration and adjustments as needed, education on donning and doffing, device care and cleaning, scalp care, (written instructions will be provided, progressive wearing schedule and monitoring logs). If consented, photography or videography may be performed to document progress at physical visits. Subject will be issued with HPPD to bring home to adapt to it.

Visit 1.1: Follow up telephone call on day 1 after visit 1 from study coordinator.

Visit 1.2: Follow up telephone call 3 days after visit 1.1 from study coordinator.

Visit 2 (end Week 1): Outpatient follow-up review and questionnaires by study coordinator.

Visit 3 (end week 2): Outpatient follow-up review and questionnaires by study coordinator.

Visit 4 (end week 4): Outpatient follow-up review and questionnaires by study coordinator.

Visit 4.5 (end week 6): Follow up telephone call on week 6 from study coordinator.

Visit 5 (week 8): *Final study visit*: Outpatient follow-up review and questionnaires by study coordinator. *

End of Study (week 8 or earlier if subject undergoes cranioplasty)

* (*if subject is scheduled for cranioplasty earlier than any of the scheduled study visits, their participation will end on the date of scheduled cranioplasty)*

** Contingency plan for Covid 19: Additional post week 8 Phone follow-up x1 by study coordinator if subject has not received cranioplasty by end week 8 follow up. (study amendment submitted 22 Aug 2020)

Post Study Follow up and Procedures

1. HPPD is returned to study coordinator if subject has undergone cranioplasty.
2. On CDOC, subject status is updated to "disenrolled"
3. Outcomes on data collection form are completed and data converted to electronic copies in password protected desktop computers.
4. Study team meets after every 5 subjects enrolled to evaluate HPPD subject response, adverse events and outcome.

a. *Discontinuation Visit and Procedures*

Subjects may withdraw voluntarily from participation in the study at any time. Subjects may also withdraw voluntarily from receiving the study intervention for any reason.
In the event of a withdrawal due to adverse event (i.e. skin breakdown), subject will immediately discontinue application of the HPPD, be given appropriate care under medical supervision until the symptoms of any adverse event resolve or the subject's condition becomes stable and subject should complete an end of study evaluation.

In the event of acute unrelated medical decompensation requiring hospitalisation, subject will cease wearing schedule for HPPD till recovered. Subject will be monitored for fitness to continue study and monitoring visits, unless eligibility criteria have changed which exclude subject from participation. (e.g. subject becomes unable to understand simple instructions, vegetative state, severe wound status, terminal medical condition with life expectancy < 6 months.

If the withdrawal is voluntary and not due to an adverse event such as skin breakdown or discomfort, subject should return the HPPD, and monitoring visits and wound management will continue till skin healed.

7. TRIAL MATERIALS

7.1. Trial Product (s)

The proposed HPPD uses biocompatible material using FDMÔNylon which has high fatigue resistance, chemical resistance with impact resistance and toughness which is free of powders. 12 Model Material. (please refer to 2 attached references from vendor)

7.2. Storage and Drug Accountability

Prostheses will be stored in a provided sealed clear box in a locked cupboard at room temperature (< 55dC) and humidity with subject code label.

8. TREATMENT

8.1. Rationale for Selection of Dose

Not applicable

8.2. Study Drug Formulations

Not applicable.

8.3. Study Drug Administration

Not applicable.

8.4. Specific Restrictions / Requirements

There are no limitations on medications, herbs, vitamins and mineral supplements (while participating in the study) and there will be no alterations or restrictions to subjects' pre-existing or concurrent medication.

8.5. Blinding

Not applicable (see 6.1)

8.6. Concomitant therapy

There will be no alterations or restrictions to subjects' pre-existing or concurrent therapies whether related to medication, rehabilitation, complementary or alternative therapies medication.

9. SAFETY MEASUREMENTS

9.1. Definitions

Define terms e.g. what would be regarded as UPIRTSO events, Serious adverse events etc..
Include details of the protocol specific reporting, procedures, including the individual responsible for each step (e.g. the Investigator, the medical monitor, etc.), how decisions will be made regarding determining relatedness and grading severity, how reports will be distributed and what follow up are required. Include specific details of reporting procedures for:

- Deaths and life-threatening events
- other SAEs
- Other adverse events

9.2. Collecting, Recording and Reporting of "Unanticipated Problems Involving Risk to Subjects or Others" – UPIRTSO events to the NHG Domain Specific Review Boards (DSRB)

UPIRTSO events refers to problems, in general, to include any incident, experience, or outcome (including adverse events) that meets ALL of the following criteria:

1. **Unexpected**
   In terms of nature, severity or frequency of the problem as described in the study documentation (eg: Protocol, Consent documents etc).

2. **Related or possibly related to participation in the research**
   Possibly related means there is a reasonable possibility that the problem may have been caused by the procedures involved in the research; and

3. **Risk of harm**
   Suggests that the research places participants or others at a greater risk of harm (including physical, psychological, economic, or social harm) than was previously known or recognized.

Reporting Timeline for UPIRTSO Events to the NHG DSRB.

1. Urgent Reporting: All problems involving local deaths, whether related or not, should be reported immediately – within 24 hours after first knowledge by the NHG investigator.

2. Expedited Reporting: All other problems must be reported as soon as possible but not later than 7 calendar days after first knowledge by the NHG investigator.

9.3. Collecting, Recording and Reporting of Serious Adverse Events (SAEs) to the Health Science Authority (HSA)

1. **For Industry sponsored Trials**
   All SAEs will be reported to HSA according to the HSA Guidance for Industry "Safety Reporting Requirements for Clinical Drug Trials."

2. **For Principal Investigator initiated Trials**
   All SAEs that are unexpected and related to the study drug must be reported to HSA.

"A serious adverse event or serious adverse drug reaction is any untoward medical occurrence at any dose that:

- Results in death.
- Is life-threatening (immediate risk of death).
- Requires inpatient hospitalization or prolongation of existing hospitalization.
- Results in persistent or significant disability/incapacity.
- Results in congenital anomaly/birth defect.
- Is a Medically important event.

Medical and scientific judgment should be exercised in determining whether an event is an important medical event. An important medical event may not be immediately life threatening and/or result in death or hospitalization. However, if it is determined that the event may jeopardize the subject and/or may require intervention to prevent one of the other adverse event outcomes, the important medical event should be reported as serious."

All SAEs that are unexpected and related to the study drug will be reported. The investigator is responsible for informing HSA no later than 15 calendar days after first knowledge that the case qualifies

for expedited reporting. Follow-information will be actively sought and submitted as it becomes available. For fatal or life-threatening cases, HSA will be notified as soon as possible but no later than 7 calendar days after first knowledge that a case qualifies, followed by a complete report within 8 additional calendar days.

9.4. Safety Monitoring Plan

Data and subject feedback will be reviewed after every 5 subjects recruited. Study coordinator will flag to study team any potential complications or negative feedback from subjects for immediate attention. Subject can call study team members in case of queries listed in Informed Consent Form.
Integrity of research data will be enforced by storage of hard copy case record forms in locked cupboards in clinics and electronic data files in password protected desktop computers or laptops.

9.5. Complaint Handling –

Complaints will be reported to the PI and actions will be disseminated to study team via e mails or Tiger text mobile phone messaging service.

10. DATA ANALYSIS

10.1. Data Quality Assurance

Data will be reviewed after every 5 subjects recruited and at midway during each subjects' outpatient phase.

10.2. Data Entry and Storage

Data will be entered into hard copy case record forms kept in locked cupboards in clinics and electronic data files will be kept in password protected desktop computers or laptops. NHG REDcap platform will be used for data capture. All hard and soft copy data collection formats will be stored for 6 years after completion of the study and thereafter disposed. Hard copies will be shredded and electronic medium will be deleted.

11. SAMPLE SIZE AND STATISTICAL METHODS

11.1. Determination of Sample Size

As this is an exploratory pilot study (n=10), no detailed sample size calculation is performed.

11.2. Statistical and Analytical Plans

a. *General Considerations:*
b. *Data will be captured using NHG Redcap platform and data will be collected on MS Excel, Windows 8 and analysed using SPSS version 22 or similar software.*
c. *Safety Analyses: as above.*

d. Interim Analyses: NA

e. Describe the types of statistical interim analyses and stopping guidelines (if any) that are proposed, including their timing. As stated in section 3.5

12. ETHICAL CONSIDERATIONS

12.1. Informed Consent

Subjects/Next of kin or legally appointed representatives will be approached prior to initiation of any study procedures, by the study team (PIs or co Is) or research coordinator. Subjects will be given copy of approved ICF and adequate time to read through and understand the procedures and ask for clarifications prior to informed consent. Consent will be taken with privacy considerations in ward screened cubicle or clinic consultation room without intrusion and to protect patient confidentiality. No coercion or force will be used during the consent process and patient will be given adequate time and options to participate.
Non-English speakers will use ICF with short form translated versions and appropriate translators if needed. Consent will be taken only by study team. In obtaining and documenting informed consent, study team will comply with the SGGCP guidelines and to the ethical principles that have their origin in the Declaration of Helsinki

12.2. IRB review

This protocol is submitted together with the associated informed consent documents to NHG DSRB.

12.3. Confidentiality of Data and Patient Records

Hard copy data will be coded with code numbers /subject initials only and the list of subjects' full names and NRIC numbers matching these codes will be kept in the investigator file located in a locked cupboard. Hard copy records will be kept in locked cupboard within locked clinic rooms and soft copy records will be kept in password-protected computers on desktops in the clinics. All electronic records related to this study will be stored in password protected laptop computers within locked clinic premises. No biological samples will be collected for this study.

13. PUBLICATIONS

As per data sharing agreements and research collaborative agreements, joint and independent publications between the involved TTSH and NNI departments will be permitted with non-identification of research subjects.

14. RETENTION OF TRIAL DOCUMENTS

Records for all participants, including CRFs, all source documentation (containing evidence to study eligibility, history and physical findings, laboratory data, results of consultations, etc.) as well as IRB records and other regulatory documentation will be retained by PI in locked cupboard within locked clinic rooms. Electronic records will be stored in password protected laptop computers located in locked clinic rooms.
Electronic soft copy data and hard copy data will be destroyed 6 years after completion of the study.

15. REFERENCES

1. Honeybul S. Decompressive craniectomy: a new complication. J Clin Neurosci. 2009 May;16(5):727-9. doi: 10.1016/j.jocn.2008.06.015. Epub 2009. Mar 3.

2. S. Bostro¨m, L. Bobinski, P. Zsigmond, and A. Theodorsson. Improved brain protection at decompressive craniectomy – a new method using Palacos+ R-40 (methylmethacrylate). Acta Neurochir (Wien) (2005) 147: 279–281. DOI 10.1007/s00701-004-0480-4

3. Honeybul S. Neurological dysfunction due to large skull defect: implications for physiotherapists. J Rehabil Med 2017; 49: 204–207

4. Livesay S. Evidenced-based nursing review of craniectomy care. DOI: 10.1161/STROKEAHA.114.006355

List of Attachments

**Appendix 1**	**Study Schedule (in ICF)**
~~**Appendix 2**~~	~~**Blood Sampling Summary**~~
**Appendix 3**	**Questionnaires used in the Trial (LOV-DCF and Screening_LOV forms)**
~~**Appendix 4**~~	~~**Laboratory Tests**~~
**Appendix 5**	**Sample Patient Information Sheet and Informed Consent Form**
