# Supplementary material for: 3D-printed external cranial protection following decompressive craniectomy after brain injury: A pilot feasibility cohort study
Source: PLoS One. 2021 Oct 28;16(10):e0258296. doi: 10.1371/journal.pone.0258296 (PMC8553164; doi:10.1371/journal.pone.0258296)
Supplement: Attachment — Submitted filename: renamed_c1d5e.docx [file pone.0258296.s006.docx]

**COMMENTS – Manuscript PONE-D-20-38286**

Title: “3D-printed external cranial protection following decompressive craniectomy after brain injury: An exploratory clinical trial”

**Important note: This review pertains only to ‘statistical aspects’ of the study and so** **‘clinical aspects’ [like medical importance, relevance of the study,** **‘clinical significance and implication(s)’ of the whole study, etc.] are to be evaluated [should be assessed] separately/independently. Further please note that any ‘statistical review’ is generally done under the assumption that (such) study specific methodological [as well as execution] issues are perfectly taken care of by the investigator(s). This review is not an exception to that and so does not cover clinical aspects {however, seldom comments are made only if those issues are intimately / scientifically related & intermingle with ‘statistical aspects’ of the study}. Agreed that ‘statistical methods’ are used as just tools here, however, they are vital part of methodology [and so should be given due importance].**

**COMMENTS:** In my opinion, since it is clarified by authors that “The authors further investigate the feasibility, safety and usability of such a parameterized approach in its application towards external customized protection, head potection prototype device (HPPD) for post-acute decompression craniectomy (DC) patients.”, the word ‘feasibility‘ {Or a ‘pilot’ (as said in section ‘Materials and Methods - (I) Study Design)} may please be included along with the present ‘Exploratory’ [An exploratory clinical trial]. As is well known, a pilot study is a small-scale preliminary study conducted in order to evaluate feasibility, duration, cost, adverse events, and improve upon the study design prior to performance of a full-scale research project.

Note that ‘Exploratory’ studies termed pilot and/or feasibility studies, are a key step in assessing the feasibility and value of progressing to an effectiveness study. Such studies can provide vital information to support more robust evaluations, thereby reducing costs and minimising potential harms of the intervention [Hallingberg, B., Turley, R., Segrott, J. *et al.* Exploratory studies to decide whether and how to proceed with full-scale evaluations of public health interventions: a systematic review of guidance. *Pilot Feasibility Stud* **4,** 104 (2018). https://doi.org/10.1186/s40814-018-0290-8].

I am sure that the authors are aware of the well-known drawbacks of a single-arm design [a type of Quasi-experimental research], and it is often said that ‘alright to have ‘single-arm design’ (before-after study) for pilot study or when that is the only possibility’, however, it is very essential to keep the limitations in mind while interpreting results. Further, note that a classical/ideal clinical trial/study needs/requires a concurrently {but similarly} handled/treated appropriately selected/chosen control/comparison parallel group/arm.

Design and other details [like Fabrication, etc.] of HPPD are alright/perfect. You may know that comparison of means from non-Gaussian distributions or when level of measurement may not ‘ratio’, for paired samples (before-after) [i.e. Two samples comparison – Paired Case] is preferred (over paired t-test) to be done by Wilcoxon’s Signed-Ranked test. Further, kindly note that presently ‘SPSS’ stands for ‘Statistical Product and Service Solutions’ [and not for Statistical Package for the Social Science any more]. Refer to page 608 of a text book ‘Medical Biostatistics’ by Indrayan & Sarmukaddam, ISBN: O-8247-0426-6, Marcel Dekkar Inc., New York, 2001.

Study Limitations [section: (iv)] are totally agreed and by statistical point of limitations 1 & 3 [(i) the small number of subjects, (ii) single centre descriptive study, (iii) lack of a control group] are causing to stop the review here. As pointed out in the ‘Important note’ above (in the beginning) this review pertains only to ‘statistical aspects’ of the study and so ‘clinical aspects’ [like medical importance, relevance of the study, ‘clinical significance and implication(s)’ of the whole study, etc.] are to be evaluated [should be assessed] separately/independently.
