# Supplementary material for: 3D-printed external cranial protection following decompressive craniectomy after brain injury: A pilot feasibility cohort study
Source: PLoS One. 2021 Oct 28;16(10):e0258296. doi: 10.1371/journal.pone.0258296 (PMC8553164; doi:10.1371/journal.pone.0258296)
Supplement: Attachment — Submitted filename: RESPONSE TO REVIEWERS_PONE_D20_38286.docx [file pone.0258296.s007.docx]

4 August 2021

Dear PLOS one Editors.

**RE: PONE-D-2038286 rebuttal**

On behalf of the authors, we thank you for the comprehensive comments to improve the quality and clarity of the manuscript and we submit a revised version with our point by point (>>) rebuttal.

Journal Requirements:

When submitting your revision, we need you to address these additional requirements.

1. Please ensure that your manuscript meets PLOS ONE's style requirements, including those for file naming. The PLOS ONE style templates can be found at
   https://imsva91-ctp.trendmicro.com:443/wis/clicktime/v1/query?url=https%3a%2f%2fjournals.plos.org%2fplosone%2fs%2ffile%3fid%3dwjVg%2fPLOSOne%5fformatting%5fsample%5fmain%5fbody.pdf&umid=3B088F6D-C85E-1D05-90DC-B99F42FB63A6&auth=6e3fe59570831a389716849e93b5d483c90c3fe4-2ae0558fb485b957534e90fb0a016cc60d768301 and 
   <https://imsva91-ctp.trendmicro.com:443/wis/clicktime/v1/query?url=https%3a%2f%2fjournals.plos.org%2fplosone%2fs%2ffile%3fid%3dba62%2fPLOSOne%5fformatting%5fsample%5ftitle%5fauthors%5faffiliations.pdf&umid=3B088F6D-C85E-1D05-90DC-B99F42FB63A6&auth=6e3fe59570831a389716849e93b5d483c90c3fe4-a6dca308f3906a6aa734d1bdce1537fc4809c02b>

>> We have complied with the title page formatting suggestions accordingly. Acknowledgements have been moved from end of manuscript to the title page.

2. Thank you for submitting your clinical trial to PLOS ONE and for providing the name of the registry and the registration number. The information in the registry entry suggests that your trial was registered after patient recruitment began. PLOS ONE strongly encourages authors to register all trials before recruiting the first participant in a study.

As per the journal’s editorial policy, please include in the Methods section of your paper:

1) your reasons for your delay in registering this study (after enrolment of participants started);

>>NCT application was drafted after DSRB approval prior to first subject recruitment but there could have been inadvertent delays in the final approval and release of NCT document to public domain (dated 7 Nov 19) resulting in this anomaly.

2) confirmation that all related trials are registered by stating: “The authors confirm that all ongoing and related trials for this drug/intervention are registered”.

>>>>We apologise for this oversight and have included his in line 151-2.

3. During our internal checks, we noted that one or more of the authors is affiliated with AuMed Pte Ltd. Please add this affiliation information to the Financial Disclosure/Conflict of Interest statement in your online submission form.

>>We have added the COI statement for Catherine Chia and Jun Cong, Looi both from AuMed Pte Ltd in the online submission portal.

4. Please note that PLOS ONE does not copy edit accepted manuscripts (https://imsva91-ctp.trendmicro.com:443/wis/clicktime/v1/query?url=https%3a%2f%2fjournals.plos.org%2fplosone%2fs%2fcriteria%2dfor%2dpublication%23loc%2d5&umid=3B088F6D-C85E-1D05-90DC-B99F42FB63A6&auth=6e3fe59570831a389716849e93b5d483c90c3fe4-45e78fe424e5003c48290a1eedb0d0db86a1f495). To that effect, please ensure that your submission is free of typos and grammatical errors.

>> We have put the manuscript through spell check
*Please also consider whether some images in your submission should be moved to the Supplemental information due to a potentially graphic nature.

>>We have obtained written informed consent from the subject featured which has been uploaded and any identifiable facial features have been occluded. The images are important to demonstrate the project outcome and feasibility.

5. We note that you have stated that you will provide repository information for your data at acceptance. Should your manuscript be accepted for publication, we will hold it until you provide the relevant accession numbers or DOIs necessary to access your data. If you wish to make changes to your Data Availability statement, please describe these changes in your cover letter and we will update your Data Availability statement to reflect the information you provide.

>> We are working with our institution on the accession numbers/DOI linking with the deidentified data file and will provide this in due course.

6. Please upload a new copy of Figure 4 and 5 as the detail is not clear. Please follow the link for more information: https://imsva91-ctp.trendmicro.com:443/wis/clicktime/v1/query?url=https%3a%2f%2fblogs.plos.org%2fplos%2f2019%2f06%2flooking%2dgood%2dtips%2dfor%2dcreating%2dyour%2dplos%2dfigures%2dgraphics%2f%22&umid=3B088F6D-C85E-1D05-90DC-B99F42FB63A6&auth=6e3fe59570831a389716849e93b5d483c90c3fe4-98fa42cf7784c7e66137e95477262f2ebb1d0f31 https://imsva91-ctp.trendmicro.com:443/wis/clicktime/v1/query?url=https%3a%2f%2fblogs.plos.org%2fplos%2f2019%2f06%2flooking%2dgood%2dtips%2dfor%2dcreating%2dyour%2dplos%2dfigures%2dgraphics%2f&umid=3B088F6D-C85E-1D05-90DC-B99F42FB63A6&auth=6e3fe59570831a389716849e93b5d483c90c3fe4-bc1149b48426822212c49002f8132262f372855d.

>> We have re attached revised figure 4-5,without changes to figure 1-3 or tables .

[Note: HTML markup is below. Please do not edit.]

Reviewers' comments:

Reviewer's Responses to Questions

**Comments to the Author**

1. Is the manuscript technically sound, and do the data support the conclusions?

The manuscript must describe a technically sound piece of scientific research with data that supports the conclusions. Experiments must have been conducted rigorously, with appropriate controls, replication, and sample sizes. The conclusions must be drawn appropriately based on the data presented.

Reviewer #1: Partly

Reviewer #2: Partly

Reviewer #3: Yes

2. Has the statistical analysis been performed appropriately and rigorously?

Reviewer #1: Yes

Reviewer #2: Yes

Reviewer #3: I Don't Know

3. Have the authors made all data underlying the findings in their manuscript fully available?

The [PLOS Data policy](https://imsva91-ctp.trendmicro.com:443/wis/clicktime/v1/query?url=http%3a%2f%2fwww.plosone.org%2fstatic%2fpolicies.action%23sharing&umid=3B088F6D-C85E-1D05-90DC-B99F42FB63A6&auth=6e3fe59570831a389716849e93b5d483c90c3fe4-74459c255102b4d2575c89a560303c7a1820c7a3) requires authors to make all data underlying the findings described in their manuscript fully available without restriction, with rare exception (please refer to the Data Availability Statement in the manuscript PDF file). The data should be provided as part of the manuscript or its supporting information, or deposited to a public repository. For example, in addition to summary statistics, the data points behind means, medians and variance measures should be available. If there are restrictions on publicly sharing data—e.g. participant privacy or use of data from a third party—those must be specified.

Reviewer #1: Yes

Reviewer #2: No

Reviewer #3: Yes

4. Is the manuscript presented in an intelligible fashion and written in standard English?

PLOS ONE does not copyedit accepted manuscripts, so the language in submitted articles must be clear, correct, and unambiguous. Any typographical or grammatical errors should be corrected at revision, so please note any specific errors here.

Reviewer #1: Yes

Reviewer #2: Yes

Reviewer #3: Yes

5. Review Comments to the Author

Please use the space provided to explain your answers to the questions above. You may also include additional comments for the author, including concerns about dual publication, research ethics, or publication ethics. (Please upload your review as an attachment if it exceeds 20,000 characters)

**Reviewer #1:**

In this work, the authors prospectively enrolled 10 craniectomy patients in a pilot trial of 3d printed HPPD as an alternative to standard helmet wearing. The work is novel, and analysis of findings based on their data is largely sound. I do have several concerns regarding the work that require clarification

1. The fundamental argument seems to be proposing an alternative to helmet wearing. However, other than in weight, there is no comparison to helmets (i.e. associated incisional breakdown, patient compliance, etc). Further, in the introduction, the authors make several claims regarding locally available helmet option shortcoming, but these require citation or the caveat of being authors anecdotal experience. I think it would also be important to compare as much as is able wearing patterns between HDDPs and helmets.
>>We thank reviewer #1 for this important comment and have clarified this in line 122-126. To date, there are no local or recognised guidelines or publications related to this.

2. The authors note that HPPDs were not administered until a minimum of 30 days after hospital discharge. Was there helmet or other protective strategies made until then?

>>We have clarified this in line 174, 164 and under inclusion criteria (iv) > 30 days post-DC; (v) healed post-DC surgical wounds, and

And the authors note a mean of 76 days between discharge and HDDP application; How does this compare to typical availability of helmets?

>>We have clarified this in line 164-166.

Does that mean that HDDPs were being donned on significantly more healed wounds than helmets?

>>We cannot comment on this as there is no local pre-study data on helmet use prior to HPPD study enrolment. The inclusion criteria included fully healed incisional wounds.

3. In inclusion criteria, vegetative state requires clarification.

>> We have clarified the “vegetative state” definition in line 180-181.

4. In discussion, the discussion of FIM mean gains in a random selection of 10 craniectomy patients I do not think adds to the understanding of HDDP usage and should be removed

>> We have removed in table 2

5. Was care taken to avoid contact with the incisions? Was pruritis or pressure in any patients observed along the incision.

>>Utmost care was taken during HPPD fabrication to relieve pressure on incisions and no incisional symptoms were noted after 30minutes of HPPD fitting or during 8 weeks of follow-up (refer to line 296-297)

How much overlap with non-resected bone was made to allow for stable HDDP support?

>>We have added the width of the overlap in line 206.

**Reviewer #2**:

Interesting study of custom printed external protection device in 10 patients s/p decompressive craniectomy. The authors state that the device is feasible and safe. I applaud the authors for their novelty in study design. I have several general and methodological comments for consideration.

- The authors state this was an "exploratory clinical trial" however there are no comparison groups or randomization. Possibly a cohort study is more appropriate?

>>We thank the reviewer for this vital comment and we have retitled the study description in line 3 141 as “A pilot feasibility cohort study”

- How were the subjects selected - convenience sample, consecutive enrolment over some period of time, ?

>>We have clarified this in line that consecutive enrolment method was used for all subjects. (lines 158 and 160)

- Methodological issues: How far did the implant overlay existing bone and how was this optimized?

>> We have added the width of the overlap and elaborated its optimization in lines 205-206.

What algorithm was used for 3-D printing?

>>We have added the important 3-D printing parameters in lines 225-226.

What material was the helmet made of and has this type of material been studied and or vetted?

>>We have elaborated on this line 227-232. The single material which the helmets were made of (FDM Nylon 12, Stratasys, USA) was mentioned in line 224. This material was selected for its printability at a fine resolution (0.1778mm) for the available machine (Fortus 450mc), its high Elongation at Break (30%, ASTM D638, Stratasys USA), low specific gravity (1.00, ASTM D648, Stratasys USA) and comparatively high tensile strength (4,600 psi, ASTM D638, Stratasys, USA). Screening was made across materials printable at the same resolution and machine.

Was there any standardization of when and how long the helmets were worn?

>>We have clarified this in line 241-249 and a gradual tolerance building till 2-3 hours of continuous wear was tolerated.

Why were there 12 helmets for 10 subjects?

>>The reason for this is stated in line 318-319: “A total of 12 HPPDs were fabricated for 10 enrolled subjects as 2 (subjects 5, 6) each received 2 HPPDs, due to a misplacement and a suboptimal fit each”

How did the authors determine 10 subjects to be a power sample sufficient for publishing on a clinical trial?

>>We have addressed this in lines 275-279: This study was shaped to be a small-scale preliminary study mainly to evaluate the feasibility and safety of HPPD prior to a full-scale research, thus a large number of subjects is not recommended before the safety of the device is ascertained. Isaac and Micheal (1995) have recommended the sample size for pilot studies to range between 10 to 30 and therefore we decided to go with the minimum required sample size. We are aware of this limitation and therefore our analyses are mainly descriptive and we have mentioned this. (Issac, S., & Michael, W.B. (1995). Handbook in Research and Evaluation. San Diego, CA: Educational and Industrial Testing Services)

Are there inherent weaknesses in the material comprising the helmet (e.g. to elements, trauma)?

>> The single material used (FDM Nylon 12, Stratasys, USA) is a nylon polymer that are susceptible to low Heat Deflection temperature (82ºC at 264 psi, ASTM D650, Stratasys, USA) which rendered itself not suitable for steam sterilization processes.

Were these patients considered for cranioplasty during the first 8 weeks?

>>We thank the reviewer for this comment and 2/10 underwent elective cranioplasty during the 8 week follow up period (lines 289). Overall, the clinical decision for cranioplasty was undecided or variable in the remaining 8 subjects, dependent on neurosurgeon decision /patient physical status or surgical fitness.

What comprised the cushion on the edges of the helmet to bone to prevent pressure injuries?

>>We have clarified this in lines 238-240.

Are long-term outcomes planned and/or evaluated?

>>The monitoring protocol was not continued after the study duration of 8 weeks, hence we did not evaluate long-term outcomes systematically. Anecdotally, 2 subjects (#1,2) under corresponding author’s follow up continued uncomplicated post-study wear of HPPD till cranioplasty in fall of 2020 and continuing wear due to late complications of seizures and COVID-19 related elective surgical list delays.

- Without a comparison group, the results are observational rather than conclusive. How do the results compare to standard recovery for patients who did not have a helmet?

>>We noted this comment and have placed this in the study limitations (lines 468-70) and are unable to comment as we did not have a comparator group. Furthermore, it was not our aim to compare recovery in helmeted and non-helmeted patients with decompression craniectomy.

- In the conclusions the authors state that the HPPD was an "acceptable external cranial protection post-DC over 8 weeks". How do the authors qualify "acceptable external cranial protection"?

>>We clarify that based on subjective ratings from 9/10 subjects in figure 4 and 5 related to wearing time, functional activities and cosmesis ratings during the follow-up phase, that there was initial good acceptability. (line 375-380)

I think the paper has potential to be publishable, however in its current state is limited by very small sample size and unclear methodology and at best would qualify as a brief report or letter to the editor.

>>We have noted this small sample in the statistical analysis (line 275) and study limitations (line 468) We can further explain that due to the novel nature of the research (risk potential), this prompted a feasibility single arm phase 1 study to allow preliminary safety and feasibility results to be established sooner prior to attempting a larger scale study. We were also challenged by limited grant funding quantum and duration of 18 months to complete the study in 2020, a COVID year. Further work is also explained in line 491 with regards to further potential.

**Reviewer #3:**

Important note: This review pertains only to ‘statistical aspects’ of the study and so ‘clinical aspects’ [like medical importance, relevance of the study, ‘clinical significance and implication(s)’ of the whole study, etc.] are to be evaluated [should be assessed] separately/independently. Further please note that any ‘statistical review’ is generally done under the assumption that (such) study specific methodological [as well as execution] issues are perfectly taken care of by the investigator(s). This review is not an exception to that and so does not cover clinical aspects {however, seldom comments are made only if those issues are intimately / scientifically related & intermingle with ‘statistical aspects’ of the study}. Agreed that ‘statistical methods’ are used as just tools here, however, they are vital part of methodology [and so should be given due importance].

COMMENTS: In my opinion, since it is clarified by authors that “The authors further investigate the feasibility, safety and usability of such a parameterized approach in its application towards external customized protection, head protection prototype device (HPPD) for post-acute decompression craniectomy (DC) patients.”, the word ‘feasibility‘ {Or a ‘pilot’ (as said in section ‘Materials and Methods - (I) Study Design)} may please be included along with the present ‘Exploratory’ [An exploratory clinical trial]. As is well known, a pilot study is a small-scale preliminary study conducted in order to evaluate feasibility, duration, cost, adverse events, and improve upon the study design prior to performance of a full-scale research project.

>>We thank the reviewer 3 for this comment and have retitled the study to reflect a pilot feasibility trial. (line 3)

Note that ‘Exploratory’ studies termed pilot and/or feasibility studies, are a key step in assessing the feasibility and value of progressing to an effectiveness study. Such studies can provide vital information to support more robust evaluations, thereby reducing costs and minimising potential harms of the intervention [Hallingberg, B., Turley, R., Segrott, J. et al. Exploratory studies to decide whether and how to proceed with full-scale evaluations of public health interventions: a systematic review of guidance. Pilot Feasibility Stud 4, 104 (2018). https://imsva91-ctp.trendmicro.com:443/wis/clicktime/v1/query?url=https%3a%2f%2fdoi.org%2f10.1186%2fs40814%2d018%2d0290%2d8&umid=3B088F6D-C85E-1D05-90DC-B99F42FB63A6&auth=6e3fe59570831a389716849e93b5d483c90c3fe4-97cd281da3405c6929b93e5f316e9b623672cacb].

I am sure that the authors are aware of the well-known drawbacks of a single-arm design [a type of Quasi-experimental research], and it is often said that ‘alright to have ‘single-arm design’ (before-after study) for pilot study or when that is the only possibility’, however, it is very essential to keep the limitations in mind while interpreting results. Further, note that a classical/ideal clinical trial/study needs/requires a concurrently {but similarly} handled/treated appropriately selected/chosen control/comparison parallel group/arm.
>>We thank reviewer 3 for this comment and have retitled accordingly to reflect a “pilot study” design (line 3) and future directions in line 476.

Design and other details [like Fabrication, etc.] of HPPD are alright/perfect. You may know that comparison of means from non-Gaussian distributions or when level of measurement may not ‘ratio’, for paired samples (before-after) [i.e. Two samples comparison – Paired Case] is preferred (over paired t-test) to be done by Wilcoxon’s Signed-Ranked test.

>> We thank the reviewer for this comment and have amended the test to Wilcoxon’s Signed Ranked test and the results and P values are similar. (line 284)

Further, kindly note that presently ‘SPSS’ stands for ‘Statistical Product and Service Solutions’ [and not for Statistical Package for the Social Science any more]. Refer to page 608 of a text book ‘Medical Biostatistics’ by Indrayan & Sarmukaddam, ISBN: O-8247-0426-6, Marcel Dekkar Inc., New York, 2001.

>> We have corrected this in line 279-280.

Study Limitations [section: (iv)] are totally agreed and by statistical point of limitations 1 & 3 [(i) the small number of subjects, (ii) single centre descriptive study, (iii) lack of a control group] are causing to stop the review here. As pointed out in the ‘Important note’ above (in the beginning) this review pertains only to ‘statistical aspects’ of the study and so ‘clinical aspects’ [like medical importance, relevance of the study, ‘clinical significance and implication(s)’ of the whole study, etc.] are to be evaluated [should be assessed] separately/independently.

>>We thank the reviewer for his comments.

6. PLOS authors have the option to publish the peer review history of their article ([what does this mean?](https://imsva91-ctp.trendmicro.com:443/wis/clicktime/v1/query?url=https%3a%2f%2fjournals.plos.org%2fplosone%2fs%2feditorial%2dand%2dpeer%2dreview%2dprocess%23loc%2dpeer%2dreview%2dhistory&umid=3B088F6D-C85E-1D05-90DC-B99F42FB63A6&auth=6e3fe59570831a389716849e93b5d483c90c3fe4-5cc09786e74453f45b3bc59d770306f77db76462)). If published, this will include your full peer review and any attached files.


If you choose “no”, your identity will remain anonymous but your review may still be made public.


**Do you want your identity to be public for this peer review?** For information about this choice, including consent withdrawal, please see our [Privacy Policy](https://imsva91-ctp.trendmicro.com:443/wis/clicktime/v1/query?url=https%3a%2f%2fwww.plos.org%2fprivacy%2dpolicy&umid=3B088F6D-C85E-1D05-90DC-B99F42FB63A6&auth=6e3fe59570831a389716849e93b5d483c90c3fe4-39999ab5b62ed09342fe580930d18bc56b032c72).

Reviewer #1: **Yes:** Michael J. Feldman

Reviewer #2: No

Reviewer #3: No

[NOTE: If reviewer comments were submitted as an attachment file, they will be attached to this email and accessible via the submission site. Please log into your account, locate the manuscript record, and check for the action link "View Attachments". If this link does not appear, there are no attachment files.]

While revising your submission, please upload your figure files to the Preflight Analysis and Conversion Engine (PACE) digital diagnostic tool, [https://pacev2.apexcovantage.com/](https://imsva91-ctp.trendmicro.com:443/wis/clicktime/v1/query?url=https%3a%2f%2fpacev2.apexcovantage.com&umid=3B088F6D-C85E-1D05-90DC-B99F42FB63A6&auth=6e3fe59570831a389716849e93b5d483c90c3fe4-2ebb42c5fa7611a91ae311547678acefa9c51695). PACE helps ensure that figures meet PLOS requirements. To use PACE, you must first register as a user. Registration is free. Then, login and navigate to the UPLOAD tab, where you will find detailed instructions on how to use the tool. If you encounter any issues or have any questions when using PACE, please email PLOS at [figures@plos.org](mailto:figures@plos.org). Please note that Supporting Information files do not need this step.

Thank you

Yours sincerely

Dr Karen Chua

Corresponding and first author
